# Genome-Wide Identification of the *NAC* Gene Family in *Brassica rapa* (L.) and Expression Pattern Analysis of *BrNAC2s*

**DOI:** 10.3390/plants14060834

**Published:** 2025-03-07

**Authors:** Weiqiang Li, Fan Ping, Huixuan Jiang, Shuqing Zhang, Tong Zhao, Kaiwen Liu, Hongrui Yu, Iqbal Hussian, Xiliang Ren, Xiaolin Yu

**Affiliations:** 1 Department of Horticulture, College of Agriculture and Biotechnology, Zhejiang University, Hangzhou 310058, China; 12216070@zju.edu.cn (W.L.); 22216177@zju.edu.cn (F.P.); 22216173@zju.edu.cn (H.J.); 22216214@zju.edu.cn (S.Z.); 12216071@zju.edu.cn (T.Z.); 12016052@zju.edu.cn (K.L.); 12416065@zju.edu.cn (H.Y.); 11816119@zju.edu.cn (I.H.); 2 Group of Vegetable Breeding, Hainan Institute of Zhejiang University, Sanya 572000, China; 3 Key Laboratory of Horticultural Plant Integrative Biology Research and Application in Zhejiang Province, Hangzhou 310058, China; 4 Institute of Vegetable Science, Ningbo Academy of Agricultural Sciences, Ningbo 315042, China; xl_ren@sohu.com

**Keywords:** *NAC* gene family, Chinese cabbage, *Brassica rapa*, flower development, expression profile

## Abstract

Flowers are one of the most important organs in plants. Their development serves as a key indicator of the transition from vegetative to reproductive growth and is regulated by various internal signals and environmental factors. NAC (NAM, ATAF, CUC) transcription factors (TFs) play a crucial regulatory role in floral organ development; however, research on the analysis and identification of the NAC TF family in Chinese cabbage (*Brassica rapa* L.) remains limited. In this study, we performed a comprehensive genome-wide analysis of *NACs* in *B. rapa* and identified 279 members of the *BrNAC* gene family. Their physicochemical properties, domain structure, collinearity relation, and *cis*-regulatory elements were evaluated. Phylogenetic analysis indicates that NAC proteins from *Arabidopsis*, *B. rapa*, *B*. *oleracea*, and *B*. *nigra* can be classified into seven distinct clades. *BrNACs* exhibit a tissue-specific expression, and nine *BrNACs* being specifically expressed in the inflorescence. Furthermore, nine flower-related *BrNACs* were selected for RT-qPCR analysis to validate their expression profiles. *BrNAC2s* has been cloned to investigate their subcellular localization, and examine the expression patterns of their promoters in *Arabidopsis* inflorescences. *BrNAC2a* and *BrNAC2c* are highly expressed in stamens while *BrNAC2b* exhibits elevated expression in pistils and pedicel. Collectively, our findings enhance the understanding of the *BrNAC* family and provide a foundation for future studies on the molecular mechanisms of *BrNACs* in floral development.

## 1. Introduction

The formation of flowers and seeds represents an adaptive advantage of plant sexual reproduction. A typical flower consists of four distinct organ types—sepals, petals, stamens, and carpels—arranged concentrically from the periphery to the center. These organs are characterized by their specific structural features and functional roles [1,2]. The four-wheel floral organs differentiate from a self-sustaining stem cell pool known as the floral meristem (FM), which originates from the peripheral regions of the shoot apical meristem (SAM). This differentiation process is governed by a multilayered regulatory network involving numerous transcriptional regulators [3,4]. Flower development is directly associated with normal flowering and fruiting processes in plants. Consequently, studying flower development holds significant theoretical and practical implications for understanding plant reproduction, enhancing crop yields, and improving crop quality [5,6].

NAC (NAM, ATAF1/2, and CUC2), one of the largest transcription factors (TFs) families in plants, is a plant-specific TF and widely present across various terrestrial plant species [7]. The first NAC TF, no apical meristem (NAM), was identified in *Petunia* in 1996 [8]. Subsequently, two other members of NAC, CUP-SHAPED COTYLEDONS (CUC) and *Arabidopsis* transcription activation factor 1/2 (ATAF1/2) were identified in *Arabidopsis* [9,10]. In recent years, members of the *NAC* family have been identified in *A. thaliana*, rice, tomato, and other plant species so far [11,12]. It is noteworthy that *NAC* family members possess a conserved DNA-binding domain of approximately 150 amino acids at the N-terminal, known as the *NAC* domain, and a variable transcriptional regulatory region at the C-terminus. This C-terminal region plays a crucial role in the transcriptional activation or inhibition of stress-responsive genes and pathways [13]. With the deepening of research on *NAC* genes, their functions have been elucidated. Most NAC TFs are primarily involved in mediating growth and development, including processes such as plant development, senescence, and morphogenesis, while also playing a significant role in the response to various stresses; the functions and underlying mechanisms of stress-related NACs have been extensively studied [13,14,15]. However, research on the analysis and identification of the NAC TF family in plant flower development remains limited to date.

Flowering is a defining characteristic of angiosperms, representing the transition from the vegetative to the reproductive growth stage. NAC TFs play crucial regulatory roles in various floral organ components and throughout the different stages of flower development in plants [15]. Several *NAC* genes have been identified as key regulatory factors in the development and maturation of floral organs [16,17,18]. Studies have shown that *NAC* related to secondary wall thickening promoting factors 1 and 2 (*NST1/2*) redundantly regulate primary cell wall thickening in the anther endothelium, facilitating anther dehiscence [16]. NAC TF At1g61110 regulates pollen wall biosynthesis through synergistic action with male sterility 1 (*MS1*) [17]. Additionally, the anther indehiscence factor (AIF) can delay anther dehiscence by inhibiting the expression of jasmonic acid biosynthesis genes [18]. CUC1 and CUC2 are important TFs in the NAC family, involved in flower organ development. They are essential for the initiation of the mating primordium and synergistically promote ovule segregation. Deletion of CUC1 and CUC2 leads to a reduction in the ovule number [19,20]. It was also found that CUCs can promote the expression of the auxin efflux carrier *PIN1*, which plays a key role in pistil development [21]. In addition, rice *OsCUC1* and *OsCUC3* mutants exhibit a decreased number of stamens and defects in leaf and filament development [22]. The post-transcriptional regulation of NAC TFs plays a particularly important role in pistil development. For example, ectopic expression of *CsNAC2* promotes female organ differentiation in cucumbers [23]. Overexpression of *AtNAC074* induces early stigma senescence and premature loss of reproductive potential, while loss-of-function mutations in *AtNAC074* extend stigma longevity [24,25]. At present, the role of NAC TFs in flower development has been preliminarily understood through phenotypic analysis; however, the regulatory mechanisms and networks of NACs in governing floral development in different species still require further investigation.

Chinese cabbage (*B. rapa* L.), which originates from northern China, is an important vegetable crop in the *Brassicaceae* family. *B. rapa* is a rich source of vitamins, fiber, and antioxidants, which promote intestinal motility. It is essential to the human diet and has become one of the most important sources of leafy vegetables and rapeseed globally [26,27]. Although NAC TF families have been identified in various plants [11,12], the functions of NACs remain poorly understood in *B. rapa*, particularly in relation to floral organ development. Fortunately, Zhang et al. [28] presented a near-complete genome assembly of *B. rapa* Chiifu v4.0 in 2023, which provide valuable tools for genomic and genetic studies in *B. rapa*.

Herein, we comprehensively identified the NAC TF members in *B. rapa* based on the v4.0 genome data. Subsequently, we performed a systematic bioinformatics analysis of the *BrNAC* gene family, including chromosome localization, phylogenetic relationships, gene structures, conserved protein motifs, collinearity features, cis-acting elements, and expression levels in various tissues. Moreover, we validated three NAC TFs specifically expressed during flower development using qRT-PCR and determined the subcellular localization of these proteins. Finally, we utilized the floral dip method to assess the expression patterns of the GUS driven by the promoters of these genes in *A. thaliana* inflorescences [29]. These results provide a foundation for further studies on the potential roles of *BrNACs* in the floral development of *B. rapa*.

## 2. Results

### 2.1. Identification of the NAC Gene Family in B. rapa

A total of 279 *NAC* genes were identified in the latest *B. rapa* genome (v4.0) using local BLAST (v2.14.0) analysis and HMM searches with the NAC protein sequences of *A. thaliana* as queries. The basic information of *BrNACs*, including gene ID, protein length, molecular weight (kDa), isoelectric point (pI), and hydropathicity, is detailed in Table S1. The predicted protein lengths range from 104 amino acids (BraA09g008570.4C.2) to 996 amino acids (BraA05g016310.4C.1), with molecular weights varying from 11.75 to 112.12 kDa. The isoelectric points span from 4.18 to 11.05, suggesting potential functional diversity in distinct microcellular environments. The instability index ranges from 23.43 to 82.47, with the majority of BrNAC proteins (71.3%) exhibiting an instability index above 40, classifying them as unstable. Additionally, grand average hydropathicity (GRAVY) analysis revealed values between −1.256 and −0.249, indicating that BrNAC proteins are predominantly hydrophilic.

**Figure 1:**
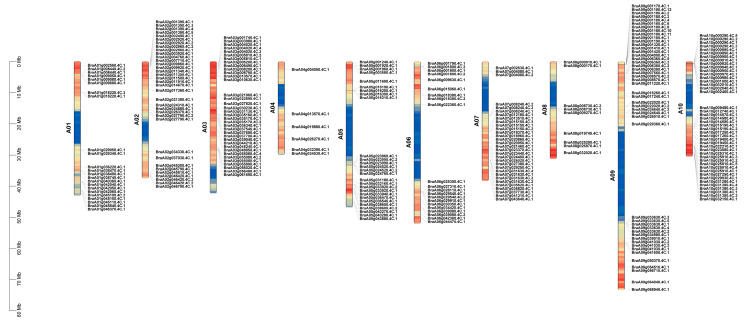
Chromosome distribution of *BrNAC* genes. The distribution of 279 *BrNAC* genes on ten *B. rapa* chromosomes.

All *BrNAC* genes are unevenly distributed across the 10 chromosomes of *B. rapa*. Chromosome A04 contains the fewest *BrNAC* genes, with only 6 members, while chromosome A09 has the highest number, with 47 genes (Figure 1). Gene duplications are considered to be one of the primary driving forces in the evolution of genomes and genetic systems [30], segmental duplication events occur frequently in the *BrNAC* genes family, with a maximum of nine duplications observed for the gene *BraA09g001180.4C* (Figure 1).

### 2.2. Sequence and Structural Analysis of BrNACs

To explore the conservation of these *BrNAC* genes, we investigated the conserved motifs via the MEME Suite, and visualized the gene structure and domains using TBtools (v2.119) [31]. As shown in Figure S1, a total of ten motifs were identified. The motif analysis revealed that proteins within the same group exhibited significant similarities, and the vast majority of *BrNACs* contained motifs 1–5 (Figure S1B), indicating a high degree of structural conservation within the *BrNAC* family. Additionally, the *BrNAC* genes possess an *NAC* domain, with gene structures varying from 0 to 9 introns. Notably, 15 *BrNAC* genes were intronless (Figure S1C,D).

### 2.3. Evolutionary Relationships of the NAC Gene Family Members

**Figure 2:**
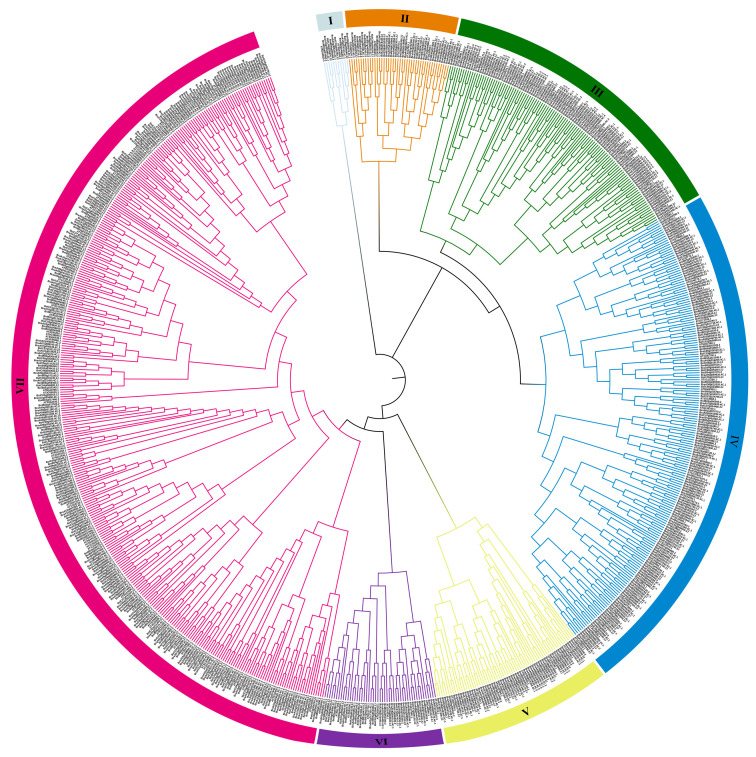
Phylogenetic analysis of NAC proteins in *B. rapa*. Phylogenetic analyses were conducted on NAC proteins from *B. rapa*, *A. thaliana*, *B. oleracea*, and *B. nigra*. MUSCLE was used for multiple sequence alignment. Phylogenetic trees were constructed using the Maximum Likelihood (ML) method with 1000 bootstrap repeats. The resulting phylogenetic tree was visualized with the online tool iTOL 6.9.1 (https://itol.embl.de/, accessed on 20 September 2024). Different colors indicate different NAC subgroups.

To investigate the evolutionary relationships of *NAC* genes across different species within the U’s triangle model, we constructed phylogenetic trees using MEGA 11.0. The analysis included four species: *B. rapa*, *A. thaliana*, *B. oleracea*, and *B. nigra*. As shown in Figure 2, the NAC proteins were categorized into seven subfamilies, with BrNAC present in all groups, demonstrating a close evolutionary relationship in the four species. We observed that Group VII contained the highest number of NAC members, including 51 AtNACs, 119 BrNACs, 95 BoNACs, and 96 BnNACs. In contrast, Group I was the smallest, consisting of only two AtNACs, three BrNACs, three BoNACs, and three BnNACs. The NAC ratios across U’s triangle species subgroups were approximately 1:1, whereas the ratios compared to *A. thaliana* ranged from 1 to 2.5. These findings suggest the presence of closely related homologous *NAC* genes between U’s triangle crops and *A. thaliana.* Given the well-characterized functions of many *AtNAC* genes, these phylogenetic groupings offer valuable insights for inferring potential functions of *BrNAC* genes based on their association with *AtNAC* genes.

### 2.4. Collinearity Analysis of BrNAC Genes with AtNAC, BoNAC, and BnNAC Genes

**Figure 3:**
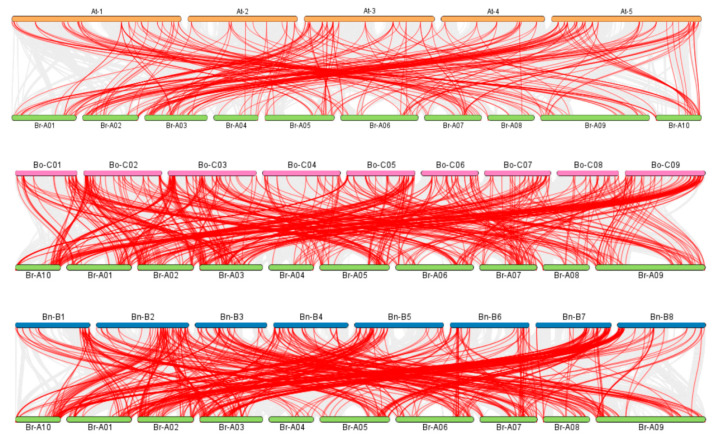
Collinear gene pair analysis of *BrNACs* between *A. thaliana*, *B. oleracea* and *B. nigra*. The red line represents collinear gene pairs. The orange color represents the *A. thaliana* chromosome, the green color represents the *B. rapa* chromosome, and the pink color represents the *B. oleracea* chromosome, the blue color represents the *B. nigra* chromosome.

To assess the collinearity relationships of the *BrNAC* gene family with *A. thaliana* and U’s triangle model species, we conducted a molecular phylogenetic analysis using the One Step MCScanX—Super Fast toolkit in TBtools (v2.119). As shown in Figure 3, the *BrNAC* gene family exhibits strong collinearity with the *NAC* genes of three other species, with the highest number of collinear gene pairs observed in *B. oleracea* (505 pairs), followed by *B. nigra* (488 pairs) and *A. thaliana* (239 pairs), suggesting a common ancestral origin and potential functional similarities across these species. Meanwhile, we also analyzed the duplication events associated with the *BrNAC* genes, and found that a large number of gene replication events occurred between *BrNACs* (Figure S2). We also performed the *Ka*/*Ks* analysis, and the results are summarized in Table S2. All duplicated *BrNAC* gene pairs exhibited *Ka*/*Ks* ratios of less than 1, suggesting that the *BrNAC* gene family has undergone purifying selection during evolution. These findings provide valuable insights into the evolutionary history and functional conservation of the *NAC* gene family, both within *B. rapa* and across different species.

### 2.5. Analysis of Cis-Elements of BrNAC Genes

**Figure 4:**
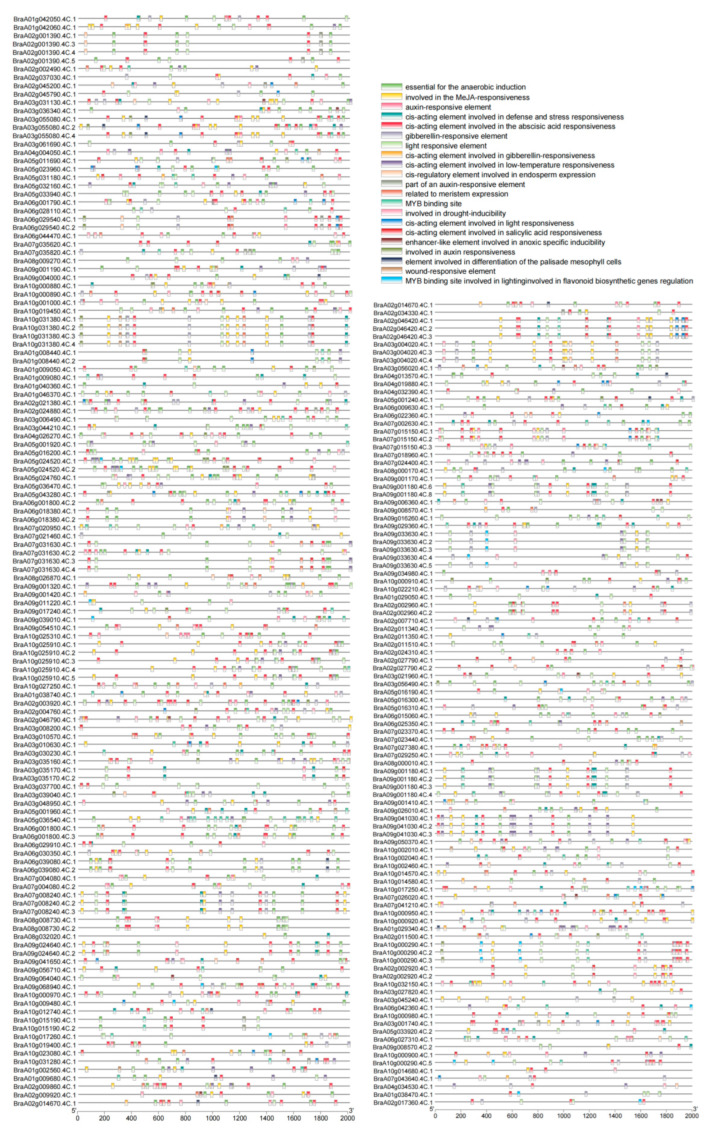
Cis-elements in the 2 kb promoter sequences of *BrNAC* genes. Different colored rectangles represent various cis-elements, with their positions indicated according to their locations within the promoters.

*Cis*-acting elements of *BrNAC* genes were analyzed using the GXF sequences program in TBtools (v2.119), the promoter sequences were obtained by determining the 2000 bp upstream sequences of the start codons of the *BrNAC* genes. The potential cis-acting elements were predicted using the PlantCARE database. We focused on five major categories of cis-acting elements, including hormone response elements, stress response elements, development-related elements, biotic and abiotic stress-related elements, and light-responsive elements (Figure 4 and Table S3). Specifically, hormone-related elements included those responsive to abscisic acid (ABA), auxin (IAA), methyl jasmonate (MeJA), and salicylic acid (SA). Growth and development-related elements were associated with meristem and endosperm expression (Figure 4). Stress-related elements encompassed responses to drought, low temperature, wounding, and pathogen defense (Figure 4). This analysis of *cis*-acting elements enhances our understanding of the potential regulatory mechanisms of *BrNAC* genes and their roles in the stress response, development, and hormone signaling in *B. rapa*.

### 2.6. Expression Profiles of BrNAC Genes in Different Tissues of B. rapa

**Figure 5:**
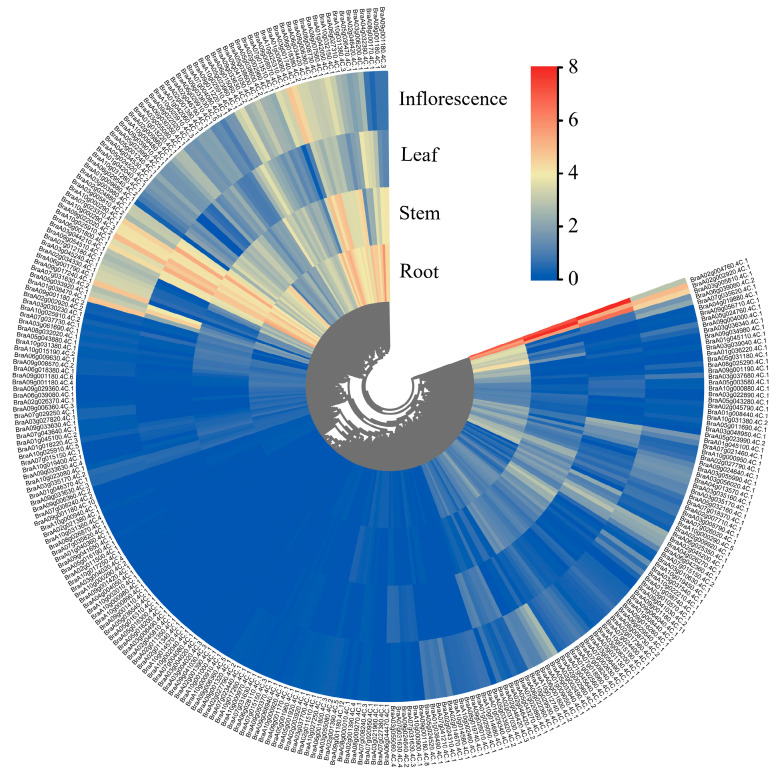
The tissue expression pattern of *BrNAC* genes in different tissues including root, stem, leaf and inflorescence. Red indicates high expression levels, and blue indicates low expression levels.

The *BrNAC* gene family plays distinct roles in plant growth and development. To further explore the functions of these genes, we examined their expression patterns across different tissues and organs using RNA-seq data. Our analysis revealed that nearly 30 *BrNAC* genes were not expressed in any of the tissues tested, suggesting that these may be non-functional pseudogenes, or alternatively, that their function is redundant in the tissues analyzed. Interestingly, several *BrNAC* genes displayed tissue-specific expression patterns (Figure 5). For instance, nearly 30 *BrNAC* genes exhibited broad expression across various tissues and organs, indicating that they may have roles in multiple biological processes. A total of 20 *BrNAC* genes were highly expressed in roots, stems, or leaves but showed low expression in the inflorescence, suggesting a potential involvement in growth and developmental processes. Additionally, nine *BrNAC* genes were expressed exclusively in the inflorescence, indicating a possible role in reproductive development. These findings provide valuable insights into the diverse functional roles and regulatory mechanisms of the *BrNAC* genes in *B. rapa*.

### 2.7. Expression Verification of Floral Development-Related BrNAC Genes

**Figure 6:**
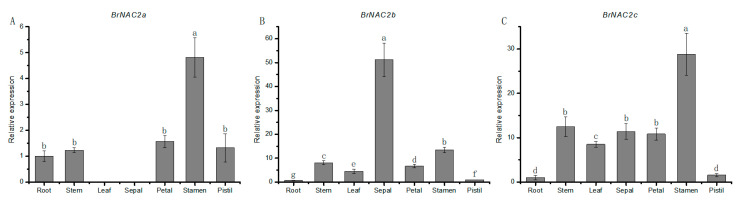
Expression profiles of *BrNAC2* genes in different tissues and organs. (**A**) Expression levels of *BrNAC2a*; (**B**) Expression levels of *BrNAC2b*; (**C**) Expression levels of *BrNAC2c.* Different letters represent significant difference (*p* < 0.05).

In this study, we utilized the BLAST search to identify direct homologous genes of *A. thaliana* for the nine *BrNAC* genes potentially involved in floral organ development (Table S4). These *BrNAC* genes were renamed based on their *Arabidopsis* counterparts. To further validate the expression profiles of these genes, RT-qPCR was performed on various floral organs of *B. rapa*. The results, presented in Figure S3 and Figure 6, revealed that with the exception of *BrNAC73* and *BrNAC78*, the remaining seven *BrNAC* genes exhibited significantly higher expression levels in different floral organs.

Previous studies have demonstrated that *AtNAC2* plays a critical role in the development and degeneration of ovule integuments [32]. Its direct homologous gene in *B. rapa* has three copies: *BrNAC2a* (*BraA03g037700.4C.1*), *BrNAC2b* (*BraA01g038470.4C.1*), and *BrNAC2c* (*BraA05g033920.4C.1*). Transcriptome results revealed that *BrNAC2b* was highly expressed in the inflorescence compared to other tissues, while the other two homologs showed no significant expression in the inflorescence (Figure 5). To validate their expression, we conducted RT-qPCR experiments to examine the expression levels of these three genes across different tissues, presenting the results as column charts (Figure 6). These results showed that all three genes were highly expressed in flower organs. Specifically, *BrNAC2a* and *BrNAC2c* exhibited significantly higher expression in stamens compared to other tissues (Figure 6A,C), while *BrNAC2b* showed the highest expression in petals (Figure 6B). These findings suggest that *BrNAC2* genes may play critical roles in the development of floral organs in *B. rapa*.

### 2.8. Subcellular Localization Analysis of BrNAC2 Proteins

**Figure 7:**
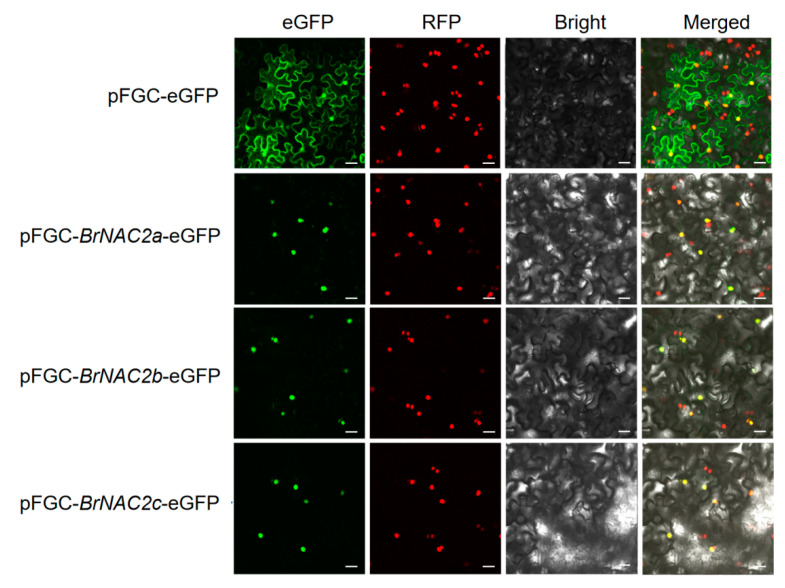
Subcellular localization of the BrNAC2s-eGFP in *Nicotiana benthamiana* leaves. pFGC5941-35S-eGFP and pFGC5941-35S-*BrNAC2s*-eGFP fusion proteins were transiently expressed in *N. tabacum* leaves. The fields included green fluorescence filed (488 nm), nucleus autofluorescence field (570 nm), bright field, and merged filed. Empty vector control showing the expression of 35S-eGFP in epidermal cells of *N. tabacum*, and co-localization of 35S-eGFP with BrNAC2 proteins observed by nucleus autofluorescence. Bars = 30 µm.

To investigate the subcellular localization of the three BrNAC2 proteins, fusion constructs (pFGC5941-*BrNAC2s*-eGFP) and a control vector (eGFP) were transiently expressed in tobacco leaf epidermal cells. Confocal laser scanning microscopy revealed that BrNAC2a, BrNAC2b, and BrNAC2c proteins were localized to the nucleus (Figure 7). These localization patterns align with the expected roles of these proteins as transcription factors, supporting their involvement in transcriptional regulation processes.

### 2.9. Expression Characteristics of BrNAC2s’s Promoters in Arabidopsis Inflorescence

**Figure 8:**
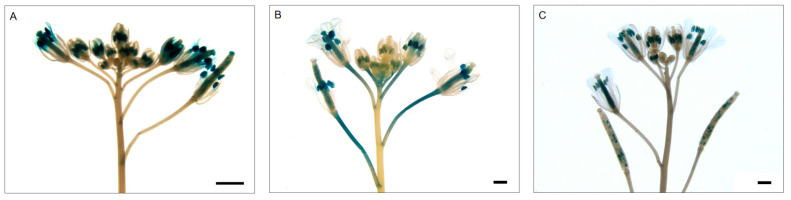
Pro*BrNAC2s*–GUS expression in transgenic *A. thaliana*. (**A**) Expression activity of *BrNAC2a* promoter in inflorescence. (**B**) Expression activity of *BrNAC2b* promoter in inflorescence. (**C**) Expression activity of *BrNAC2c* promoter in inflorescence. Scale bars = 1 mm.

To explore the spatiotemporal expression profiles of *BrNAC2*’s promoters in the inflorescence, we transformed pro*BrNAC2s*::GUS constructs into *A. thaliana* and performed GUS staining. The results revealed that the promoters of *BrNAC2a*, *BrNAC2b*, and *BrNAC2c* were capable of activating GUS expression in the inflorescence, with the strongest signals observed in the stamens and pistils (Figure 8). Specifically, *BrNAC2a* and *BrNAC2c* are highly expressed in stamens while *BrNAC2b* exhibits elevated expression in pistils and pedicel. These findings suggest that *BrNAC2* genes might potentially function as regulatory factors in the developmental processes of both pistil and stamen.

## 3. Discussion

The NAC TF family is a key regulator of floral development and one of the most extensively studied groups of transcription factors [33]. While the functions of NAC TFs in floral organogenesis have been examined in numerous species, their roles in *B. rapa* remain unexplored. Here, we identified and performed a comprehensive bioinformatics analysis of 279 *NAC* gene family members in *B. rapa* ‘Youqing 49’. We analyzed their physicochemical properties, chromosome localization, gene structure, conserved motifs, phylogenetic relationships, collinearity, and cis-acting elements. Additionally, we investigated the subcellular localization and inflorescence-specific expression profiles of three *BrNAC* genes implicated in floral development. These findings provide valuable insights into the regulatory mechanisms of *NAC* genes in *B. rapa*, laying the foundation for further functional studies.

We identified 279 *NAC* genes in the *B. rapa* genome (v4.0), significantly exceeding the number reported in Chen’s research [34]. This discrepancy is attributed to the more comprehensive nature of the *B. rapa* genome v4.0, which represents a near-complete genome assembly, compared to the partial genome (v1.5) utilized in previous research [28]. Gene structure analysis revealed variability among *BrNAC* gene family members, while those within the same subclass exhibited consistent structural patterns (Figure S1). In addition to highly conserved motifs shared across the family, *BrNAC* genes also contained five subclass-specific motifs. Members of the same subclass were characterized by similar motif patterns (Figure S1). One of the drivers of gene family expansion is the splicing and insertion of fundamental genetic fragments [35]. Variations in sequence length, domain localization, and exon count were observed among members of the same *BrNAC* family subgroup, likely resulting from splicing and fragment insertion events during the evolution of the *BrNAC* gene family. These findings suggest that the structural diversity of *BrNAC* genes may underlie their complex and specialized functions in plants.

Understanding phylogenetic relationships among species forms the foundation of many biological studies, and a precise phylogenetic tree is critical for elucidating evolutionary connections [36]. In the present study, *NAC* genes from *A. thaliana* and other two species of the U’s triangle model plants were classified into seven distinct groups. The ratio of *NAC* genes among different U’s triangle species in each subgroup was approximately 1:1, while the ratio of *B. rapa NACs* to *A. thaliana* *NACs* ranged from 1 to 2.5. These findings align with Chen et al. [34]. After the *Arabidopsis*-*Brassiceae* divergence, a whole-genome triplication (WGT) event occurred, resulting in the division of the *B. rapa* genome into three subgenomes. Consequently, *NAC* genes in *B. rapa* often have one–three orthologs [37]. This WGT event may have contributed to functional redundancy among *NAC* genes in *B. rapa*. Homologous genes, which perform similar functions across species, understanding the collinear relationships between different species will be important to future studies of gene function and evolution in plant [38,39]. Here, *BrNAC* genes exhibited strong collinearity among *A. thaliana*, *B. oleracea*, and *B. nigra*, all members of the *Brassicaceae* family. These findings suggest that most *NAC* gene family members in these species evolved from a common ancestor.

Cis-acting elements are DNA sequences located upstream of genes that play a crucial role in regulating gene expression. Studying these elements is essential for enhancing our understanding of gene regulation mechanisms [40]. In this study, we predicted the cis-acting elements of the *BrNAC* gene members, identifying various categories, including hormone-responsive elements, stress-responsive elements, development-related elements, biotic and abiotic stress-related elements, and light-responsive elements (Figure 4). Previous studies have demonstrated that environmental conditions, development-related genes, hormones, and other factors can regulate *NAC* gene expression, thereby balancing plant growth and development [41,42,43,44]. Our investigation of cis-acting elements in the *BrNAC* gene family provides valuable insights into their functional roles in plant growth, development, and defense responses, contributing to a deeper understanding of their regulatory mechanisms.

In this study, transcriptome data from various tissues of *B. rapa* were analyzed to investigate the expression patterns of the *BrNAC* gene family members. The majority of *BrNAC* genes displayed tissue-specific expression, with only a few *BrNACs* exhibiting high expression levels across multiple tissues. These findings are consistent with those of Chen’s research [34]. Whole genome duplication (WGD) results in genome-wide redundancy, which contributes to increased genetic diversity and the divergence in gene expression patterns is often the initial step in the functional divergence between duplicated genes [45,46]. Our subcellular localization analyses revealed that these proteins are predominantly localized in the nucleus. RT-qPCR analysis of floral organ expression patterns for *BrNAC2* genes demonstrated high expression levels in stamens and petals. Additionally, promoter activity assays indicated that the promoters of *BrNAC2a*, *BrNAC2b*, and *BrNAC2c* showed similar activity, with the strongest activation in pollen, followed by pistils, with lower expression observed in petals. Studies have shown that *NAC* FTs play pivotal roles in floral development, including petal fusion, ovule number regulation, and anther dehiscence [47,48]. The above results indicate the functions of *BrNAC2a*, *BrNAC2b*, and *BrNAC2c* may have been preserved throughout the evolution of the *B. rapa* genome, and these genes maintain essential roles; alternatively, these genes may have evolved to cooperate in a synergistic manner, jointly contributing to the regulation of floral development *B. rapa*.

Flowering represents a pivotal stage in the plant life cycle, marking the transition to reproductive development. This process is intricately regulated by numerous endogenous and exogenous factors, which collectively influence flower formation and development [49]. Understanding the regulatory networks governing flower development is essential, as this process directly influences agricultural yields and biomass production. The findings of this study provide a foundation for exploring the roles and regulatory mechanisms of the *BrNAC* gene family in the flowering process of *B. rapa*. Future research should incorporate phenotypic analyses to deepen our understanding of the specific contributions of *BrNAC* family members to floral development and their broader implications for crop improvement.

## 4. Materials and Methods

### 4.1. Plant Materials and Growth Conditions

*B. rapa* ‘Youqing 49’ were used as plant materials. Seeds were germinated and then sown in plastic seedling trays, the plants were grown in a climate chamber (16/8 h light/dark, 25/22 °C day/night temperature, 60–70% relative humidity, 300 µmol·m^−2^·s^−1^ photosynthetic photon flux density) consisting of a 3:1:1 mixture of nutrient soil, vermiculite, and perlite for 6–8 weeks until flowering.

### 4.2. Identification and Phylogenetics of BrNAC Family Genes in B. rapa

The genome information and related annotation files of *B. rapa* were downloaded from the Genome Database for *Brassicaceae* (www.brassicadb.cn/, accessed on 19 May 2024). The Hidden Markov Model (HMM) for the *NAC* domain (PF02365) was obtained from the Pfam database (http://pfam-legacy.xfam.org/, accessed on 21 May 2024). AtNAC protein sequences were downloaded from TAIR database (http://www.arabidopsis.org/, accessed on 25 May 2024). Firstly, we employed HMM (v3.3.2) software to search for BrNAC in the *B. rapa* protein database (E-value < 1 × 10^−5^). Secondly, the BLASTP method was used with the AtNAC protein sequence and *B. rapa* protein sequence (E-value < 1 × 10^−5^). Finally, the shared sequences obtained by the two search tools served as candidate *BrNAC* gene family members. Then, candidate BrNAC protein sequences were submitted to the NCBI-CDD (https://www.ncbi.nlm.nih.gov/Structure/bwrpsb/bwrpsb.cgi, accessed on 16 July 2024) to identify the conserved *NAC* domain.

In addition, the basic physicochemical properties, such as protein length, molecular weight (kDa), isoelectric point (pIs), and hydropathicity of BrNACs, were analyzed using ExPASy website (https://web.expasy.org/compute_pi/, accessed on 20 July 2024).

### 4.3. Chromosomal Distribution, Gene Structure, and Conserved Motifs Analysis of BrNACs

Based on information from the *B. rapa* annotation files, the chromosomal distribution and gene structure of *BrNAC* genes were visualized using TBtools (v2.119). The conserved motifs of *BrNACs* were predicted by MEME Suite v5.5.5 (https://meme-suite.org/meme/tools/meme, accessed on 25 July 2024) and performed by TBtools (v2.119) [31].

### 4.4. Phylogeny and Collinearity Analysis of BrNACs

A total of 114 *NAC* genes from *A. thaliana* (*AtNACs*), 279 from *B. rapa* (*BrNACs*), 226 from *B. oleracea* (*BoNACs*), and 226 from *B. nigra* (*BnNACs*) were utilized to construct a phylogenetic tree. *Brassicaceae* database accession numbers and the corresponding gene classification groups are presented in Table S5. Phylogenetic relationships were inferred using the Maximum Likelihood (ML) method implemented in MEGA v11.0, with 1000 bootstrap replicates.

Collinear gene pairs between *BrNAC* and *AtNAC*, *BoNAC*, and *BnNAC* genes were identified using TBtools (v2.119). Additionally, segmental and tandem duplications among *BrNAC* genes were analyzed and visualized using TBtools (v2.119) (Figure S2).

### 4.5. Analysis of Cis-Acting Elements in BrNACs’ Promoters

PlantCARE website (http://bioinformatics.psb.ugent.be/webtools/plantcare/html/, accessed on 3 August 2024) was used to predict the cis-acting elements of 2000 bp sequences upstream of the start codon of *BrNACs* obtained from *B. rapa* [50], and the subsequent results were illustrated using TBtools (v2.119).

### 4.6. RNA-Seq and RT-qPCR Analysis

The samples of root, stem, leaf, and inflorescence from *B. rapa* plants in bloom were collected, with tissues immediately frozen in liquid nitrogen and stored at −80 °C for subsequent analysis. Whole transcriptome sequencing was carried out by MK-BIO Bio-Tech (Hangzhou, China). The heatmap of *BrNAC* gene expression was generated using TBtools (v2.119). The data corresponding to the analysis are presented in Table S6.

Total RNA was extracted using the Plant RNA Extraction Kit (TRIeasy Total RNA Extraction Reagent, Shanghai, China) and reverse transcribed into cDNA using the Reverse Transcription Kit (ABScript III RT Master Mix for qPCR with gDNA Remover, Wuhan, China). Quantitative real-time PCR (qRT-PCR) was performed on a Bio-Rad CFX96 Real-Time PCR Detection System (Bio-Rad Laboratories, Inc., Hong Kong, China) with Hieff UNICON^®^ Universal Blue qPCR SYBR Green Master Mix (Yeasen, Shanghai, China). The relative expression levels of *BrNAC2* genes were calculated using the 2^−ΔΔCt^ method [51]. *BrUBC10* was used as the reference gene [52], and the primer sequences are listed in Table S7.

### 4.7. Subcellular Localization Assay of BrNAC2s Proteins

The full-length sequences of *BrNAC2a*, *BrNAC2b*, and *BrNAC2c* genes which contained arms homologous to the expression vector were amplified using the cDNA of wild-type “Youqing 49” flower bud as a template. Then, the sequences were cloned into the *Bam*H I and *Xba* I sites of the pFGC5941-eGFP vector through homologous recombination. The gene cloning primer sequences are listed in Table S8.

The plasmids were introduced into *Agrobacterium tumefaciens* (strain GV3101) and injected into 4-week-old *N. benthamiana* leaves. Photographic observations of the tobacco leaf epidermal cells were carried out in dark culture for 36–48 h after transfection on a Nikon confocal laser scanning microscope (A1-SHS) (Nikon, Tokyo, Japan). For GFP fluorescence and nuclear autofluorescence analysis.

### 4.8. ProBrNAC2s-GUS Construction and GUS Staining

To generate the *ProBrNAC2s-GUS* construct, approximately 2000 bp fragments upstream of the ATG start codon of *BrNAC2a*, *BrNAC2b*, and *BrNAC2c* were amplified using specific primers listed in Table S9. These fragments were inserted into the *Hind* III and *Xba* I sites of the *pCAMBIA1300* vector to fuse with the β-glucuronidase (GUS) reporter gene. The resulting vector was transformed into *A. thaliana* using the floral dip method [53]. To assess GUS activity, inflorescences from transgenic *Arabidopsis* plants were stained with GUS buffer (Coolaber, Beijing, China) overnight at 37 °C. The tissue was then decolorized in an ethanol gradient and photographed to visualize GUS expression.

### 4.9. Statistical Analysis

All experiments in this study were performed with three independent biological replicates. Data are expressed as the standard error (SE) of the mean. Statistical differences between groups were assessed using one-way analysis of variance (ANOVA), with significant differences indicated by different letters.

## 5. Conclusions

In summary, we performed a genome-wide identification of *NAC* genes in *B. rapa* and conducted a comprehensive analysis of their physicochemical properties, gene structures, phylogenetic relationships, collinearity, *cis*-regulatory elements, and tissue expression patterns. We also characterized the expression profiles of *BrNAC2* genes in different floral organs. Notably, *BrNAC2a* and *BrNAC2c* are highly expressed in stamens while *BrNAC2b* exhibits elevated expression in pedicel. Additionally, we investigated the activity of their promoters in *Arabidopsis* inflorescences. Our findings provide valuable insights into the role of *BrNAC* genes in floral development and lay the groundwork for future studies on their molecular mechanisms.

## Data Availability

All data in this study can be found in the manuscript or the Supplementary Materials.

## Supplementary Materials

The following supporting information can be downloaded at: https://www.mdpi.com/article/10.3390/plants14060834/s1, Figure S1: Analysis of phylogenetic relationships, motifs, domain, and gene structure of *BrNACs*; Figure S2: The duplication events analysis of *BrNACs*; Figure S3: Expression profiles of 8 *BrNAC* genes in different tissues and organs; Table S1: Physicochemical properties of *NAC* family gene in *B. rapa*; Table S2: *Brassicaceae* database accession number of genes used to construct the phylogenetic tree; Table S3: qRT-PCR primer sequences; Table S4: Primers list for subcellular localization; Table S5: Primers list for vector construction; Table S6. The data utilized for heatmap construction of *BrNAC* genes expression; Table S7. qRT-PCR primer sequences; Table S8. Primers list for subcellular localization; Table S9. Primers list for vector construction.
