# Supplementary material for: Genome-Wide Identification of the NAC Gene Family in Brassica rapa (L.) and Expression Pattern Analysis of BrNAC2s
Source: Plants (Basel). 2025 Mar 7;14(6):834. doi: 10.3390/plants14060834 (PMC11946662; doi:10.3390/plants14060834)
Supplement: Supplementary file 1 [file plants-14-00834-s001.zip › Supplementary Figures.pdf]

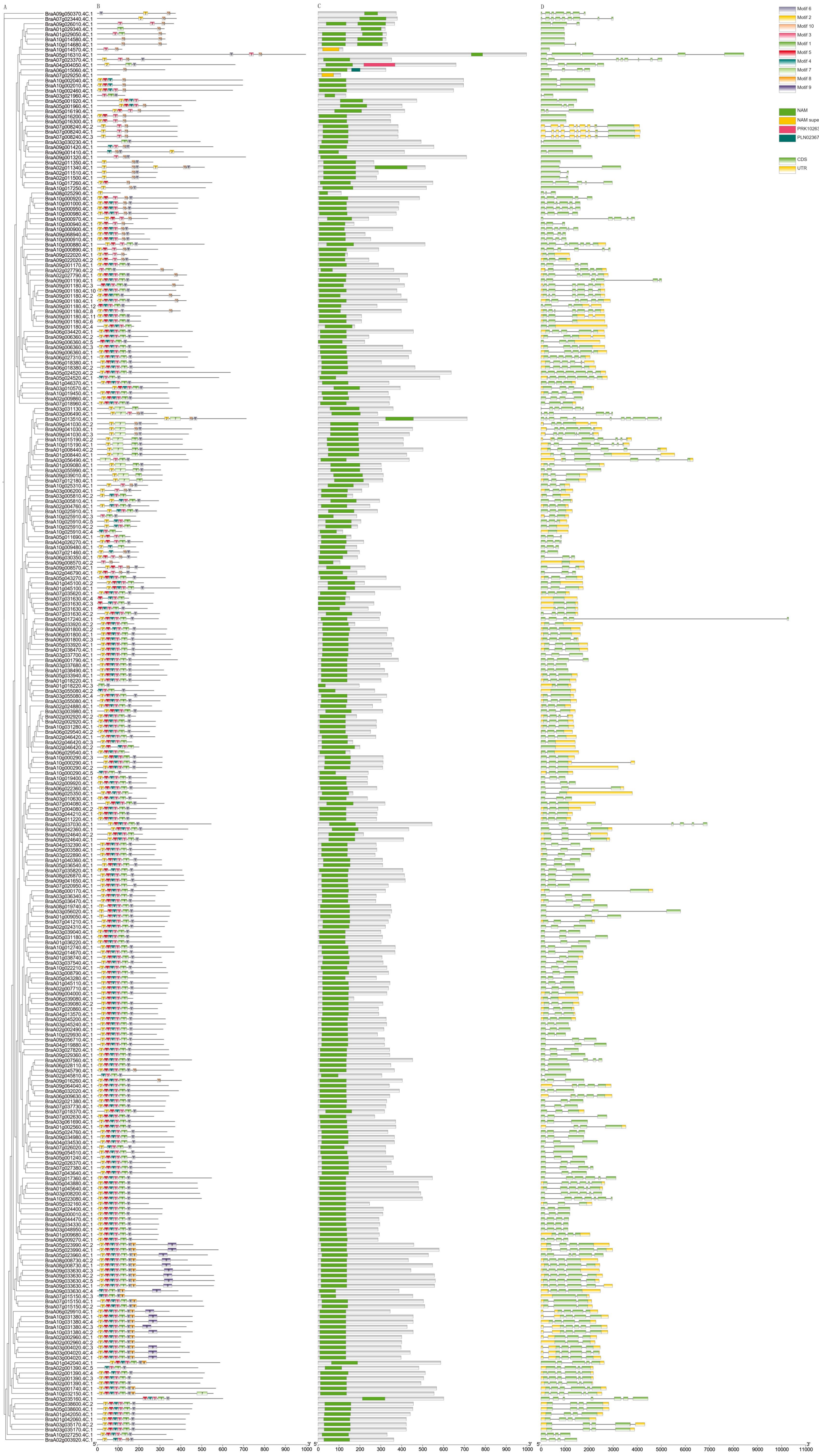

Figure S1. Analysis of phylogenetic relationships, motifs, domain and gene structure of *BrNACs.* (A) Phylogenetic tree of 279 *BrNACs*. (B) Conserved motifs arrangements of *BrNACs*. The motifs are highlighted in various colored boxes. (C) Conservative domain prediction of the *BrNACs*. Various colored boxes represents different domains. (D) Exon-intron organizations of *BrNACs*. Green boxes represents exons; black lines indicate introns.

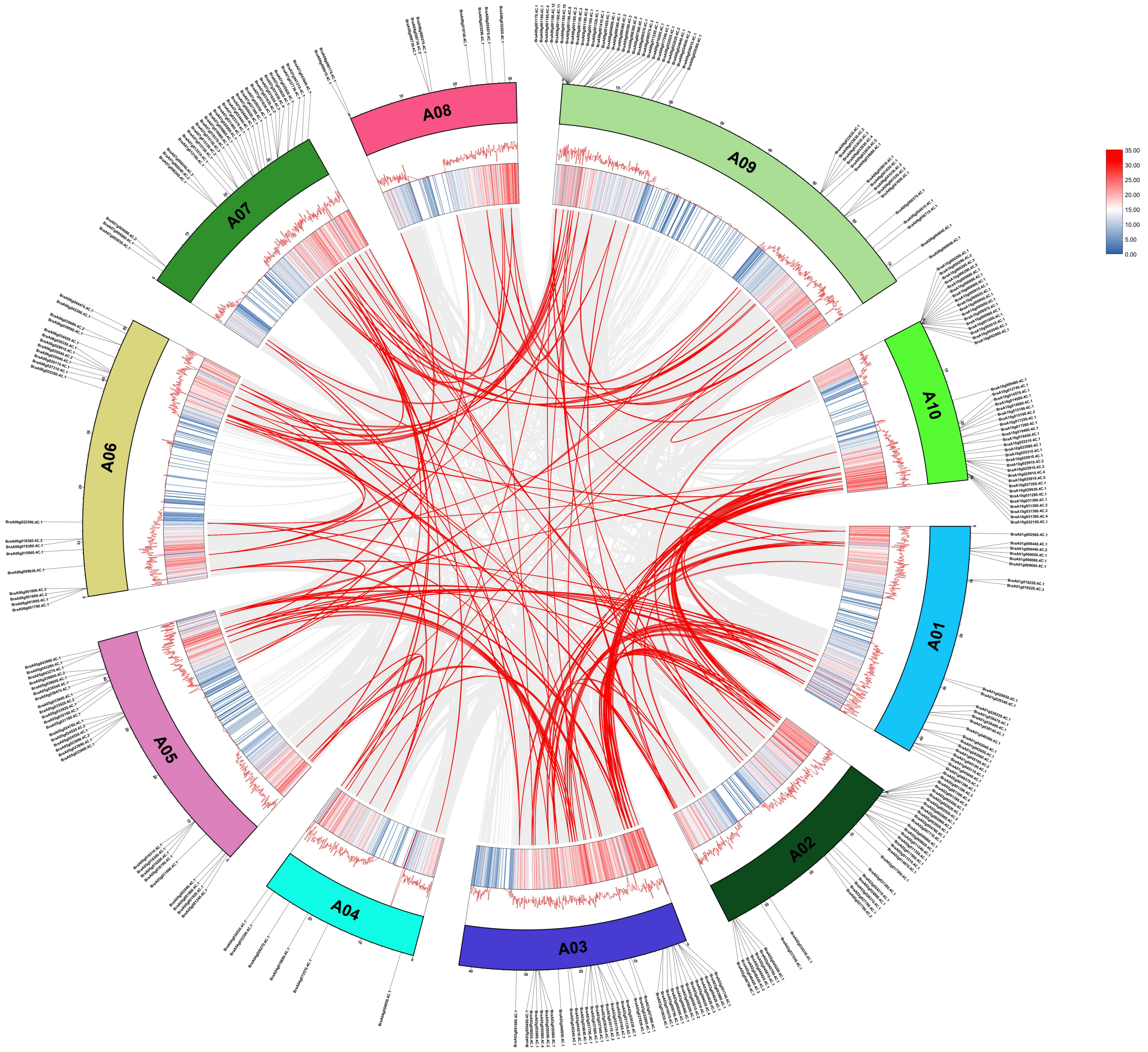

Figure S2. The duplication events analysis of *BrNACs*. Red lines represent duplicated *NAC* gene pairs; the chromosome number is shown inside each chromosome.

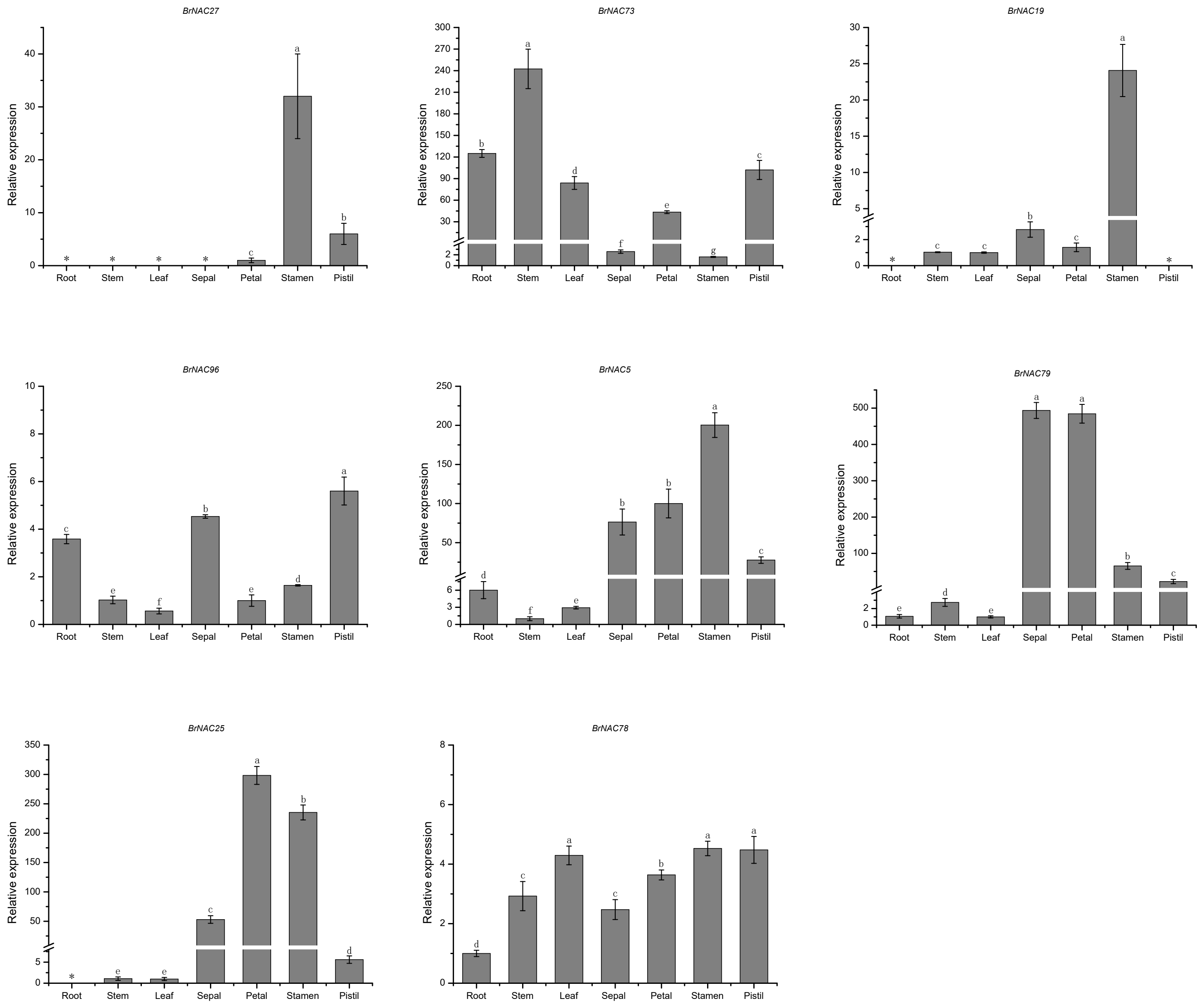

Figure S3. Expression profiles of 8 *BrNAC* genes in different tissues and organs. The * indicates that no expression was detected. Different letters represent significant difference ($p < 0.05$).
